# *GBA* and *APOE* Impact Cognitive Decline in Parkinson’s Disease: A 10-Year Population-Based Study

**DOI:** 10.1002/mds.28932

**Published:** 2022-02-02

**Authors:** Aleksandra A. Szwedo, Ingvild Dalen, Kenn Freddy Pedersen, Marta Camacho, David Bäckström, Lars Forsgren, Charalampos Tzoulis, Sophie Winder-Rhodes, Gavin Hudson, Ganqiang Liu, Clemens R. Scherzer, Rachael A. Lawson, Alison J. Yarnall, Caroline H. Williams-Gray, Angus D. Macleod, Carl E. Counsell, Ole-Bjørn Tysnes, Guido Alves, Jodi Maple-Grødem, Parkinson’s Incidence Cohorts Collaboration

**Affiliations:** 1 The Norwegian Center for Movement Disorders, Stavanger University Hospital, Stavanger, Norway; 2 Department of Chemistry, Bioscience and Environmental Engineering, University of Stavanger, Stavanger, Norway; 3 Department of Research, Section of Biostatistics, Stavanger University Hospital, Stavanger, Norway; 4 Department of Neurology, Stavanger University Hospital, Stavanger, Norway; 5 Department of Clinical Neurosciences, University of Cambridge, Cambridge, UK; 6 Department of Clinical Science, Neurosciences, Umeå University, Umeå, Sweden; 7 Department of Neurology, and Department of Neuroscience, Yale University School of Medicine, New Haven, Connecticut, USA; 8 Department of Neurology, Haukeland University Hospital, University of Bergen, Bergen, Norway; 9 Department of Clinical Medicine, University of Bergen, Bergen, Norway; 10 Biosciences Institute, Newcastle University, Newcastle upon Tyne, UK; 11 Wellcome Centre for Mitochondrial Research, Newcastle University, Newcastle upon Tyne, UK; 12 Neurobiology Research Center, School of Medicine, Sun Yat-sen University, Shenzhen, Guangdong, China; 13 Center for Advanced Parkinson Research, Harvard Medical School, Brigham and Women’s Hospital, Boston, USA; 14 Translational and Clinical Research Institute, Newcastle University, Newcastle upon Tyne, UK; 15 Institute of Applied Health Sciences, University of Aberdeen, Aberdeen, UK

**Keywords:** Parkinson’s disease, dementia, cognitive decline, *APOE*, *GBA*

## Abstract

**Background:**

Common genetic variance in apolipoprotein E (*APOE*), β-glucocerebrosidase (*GBA*), microtubule-associated protein tau (*MAPT*), and α-synuclein (*SNCA*) has been linked to cognitive decline in Parkinson’s disease (PD), although studies have yielded mixed esults.

**Objectives:**

To evaluate the effect of genetic variants in *APOE*, *GBA*, *MAPT*, and *SNCA* on cognitive decline and risk of dementia in a pooled analysis of six longitudinal, non-selective, population-based cohorts of newly diagnosed PD patients.

**Methods:**

1002 PD patients, followed for up to 10 years (median 7.2 years), were genotyped for at least one of *APOE*-ε4, *GBA* mutations, *MAPT* H1/H2, or *SNCA* rs356219. We evaluated the effect of genotype on the rate of cognitive decline (Mini-Mental State Examanation, MMSE) using linear mixed models and the development of dementia (diagnosed using standardized criteria) using Cox regression; multiple comparisons were accounted for using Benjamini–Hochberg corrections.

**Results:**

Carriers of *APOE*-ε4 (n = 281, 29.7%) and *GBA* mutations (n = 100, 10.3%) had faster cognitive decline and were at higher risk of progression to dementia (*APOE*-ε4, HR 3.57, *P* < 0.001; *GBA* mutations, HR 1.76, *P* = 0.001) than non-carriers. The risk of cognitive decline and dementia (HR 5.19, *P* < 0.001) was further increased in carriers of both risk genotypes (n = 23). No significant effects were observed for *MAPT* or *SNCA* rs356219.

**Conclusions:**

*GBA* and *APOE* genotyping could improve the prediction of cognitive decline in PD, which is important to inform the clinical trial selection and potentially to enable personalized treatment

## Introduction

Patients with Parkinson’s disease (PD) are more likely to experience cognitive decline than healthy older adults.^1^ Problems with cognition affect patients’ ability to work and function independently, placing them at higher risk of poor quality of life and nursing home placement.^2^ The evolution of cognitive symptoms in PD is very heterogeneous^1^ and impedes the recruitment of relevant participants into clinical trials. Hence, the means to identify patients at high risk of cognitive deficits would significantly improve the design and costs of future trials.

Genetic factors are candidate predictors of cognitive decline and dementia in PD (PDD), although heterogeneity in the design of published studies has contributed to inconsistent findings.^3^ Among the strongest candidates are variants in the apolipoprotein E (*APOE*), β-glucocerebrosidase (*GBA*), microtubule-associated protein tau (*MAPT*), and α-synuclein (*SNCA*) loci. The ε4 allele of *APOE* (*APOE*-ε4) is the strongest genetic risk factor for sporadic Alzheimer’s disease (AD)^4^ and the top hit in genome-wide association studies for dementia with Lewy bodies (DLB),^5^ and several large studies showed its impact on the progression of cognitive decline in PD.^6–8^ *GBA* mutations are the commonest genetic risk factors for PD,^9^ and the risk of dementia in carriers of *GBA* mutations is modulated by the type of mutation.^10^ The H1 *MAPT* haplotype has been linked to tauopathies including AD and also to PD, and common variants in *SNCA* are established risk factors for sporadic PD.^11,12^ Some studies suggest that variants in *SNCA* and *MAPT* might also affect the cognitive decline in PD,^13,14^ although results are inconsistent.^7,15,16^

Despite extensive literature on the impact of genetic variants in *APOE*, *GBA*, *MAPT*, and *SNCA* on cognitive progression in PD, large studies with prospective follow-up from the time of PD diagnosis are scarce and many studies track patients solely from a clinical environment. In this work we establish the significance of the *APOE*, *GBA*, *MAPT*, and *SNCA* loci on global cognitive decline and the development of dementia over the natural course of PD in the Parkinson’s Incidence Cohorts Collaboration (PICC). Together the six longitudinal, population-based European cohorts form a large sample of patients with deeply characterized disease progression up to 10 years from the time of diagnosis with PD.

## Methods

### Subjects

We used data from PICC, a project pooling data from six PD population-based cohorts in Northern Europe, each designed to collect demographic and clinical data at the point of diagnosis and during prospective follow-up. Each cohort is summarized in Supplementary Table 1 and has been described in detail: Cambridgeshire Incidence of Parkinson’s disease from General Practitioner to Neurologist (CamPaIGN),^17^ Incidence of Cognitive Impairment in Cohorts with Longitudinal Evaluation-PD (ICICLE-PD),^18^ New Parkinson Patient in Umeå (NYPUM),^19^ ParkWest,^20^ Parkinsonism: Incidence, Cognition and Non-motor heterogeneity in Cambridgeshire (PICNICS),^21^ and Parkinsonism Incidence in Northeast Scotland (PINE).^22^ Briefly, patients were diagnosed with idiopathic PD using UK Parkinson’s Disease Society Brain Bank criteria without using family history as an exclusion criterion. Only those with a confirmed clinical diagnosis at their last clinical visit or autopsy are eligible for the PICC study, which currently has 1107 patients (1035 [93.5%] incident cases); of these, DNA or genetic data were available for 1002 (932 [93.0%] incident cases).

### Demographic and Clinical Assessment

Data acquisition has been described in detail.^17–22^ Age at PD diagnosis, age at symptom onset, and age at baseline were defined as the age when PD diagnosis was made, at first self-reported motor symptoms, and at inclusion in the study, respectively. Patients are reassessed at regular follow-up visits (Supplementary Fig. 1). Home visits and/or telephone follow-up were offered to minimize attrition bias. The progression and severity of parkinsonism were evaluated using the Hoehn and Yahr scale^23^ and the Unified Parkinson’s Disease Rating Scale (UPDRS)^24^ Part III (CamPaIGN, NYPUM, ParkWest, PINE) or the Movement Disorders Society-UPDRS^25^ (MDS-UPDRS) Part III (ICICLE-PD, PICNICS). UPDRS-III scores were converted into MDS-UPDRS-III as described.^26^ Global cognitive function was assessed using the Mini-Mental State Examination^27^ (MMSE) scale. Dementia was diagnosed according to Diagnostic and Statistical Manual of Mental Disorders, 4th Edition^28^ (PINE, CamPaIGN, PICNICS) or Movement Disorder Society criteria^29^ (ICICLE-PD, NYPUM, ParkWest) (Supplementary Table 2).

### Genetic Data Collection

Genetic data were available or acquired for this study for *APOE*, *GBA*, *MAPT*, and *SNCA* using a combination of whole exome sequencing, genotyping arrays, or targeted genotyping using TaqMan genotyping assays. Full details are given in Supplementary methods and the final genetic data set is summarized in Supplementary Table 3.

### Statistical Analysis

For primary analysis, patients were grouped by genotype based on previous studies: *APOE*, carriers of ε4 allele versus non-carriers^6^; *GBA*, carriers of any *GBA* mutation versus non-carriers^6^; *MAPT*, carriers of H1/H1 versus H2 haplotype^14^; and *SNCA* rs356219, carriers of GG genotype versus A-allele.^30^ Primary analyses were corrected for multiple comparisons using the Benjamini–Hochberg false discovery rate (FDR) method at FDR < 0.05. For secondary analysis, carriers of *APOE*-ε4 were subdivided into carriers of one or two ε4 alleles, and *GBA* carriers were subdivided into carriers of PD risk or mild mutations, severe mutations, or variants of unknown significance. The classification of *GBA* mutations was based on pathogenicity in Gaucher disease (GD)^31^ and PD (Supplementary Table 4).

Baseline genotype-group comparisons were performed in IBM SPSS 26.0 (Armonk, NY, USA) using *t*-tests, Mann–Whitney *U* tests, or χ^2^ tests as appropriate, followed by multivariate linear regression for significant findings: age variables were compared with adjustment for study cohort and sex. Median (interquartile range [IQR]) follow-up time and cumulative proportion of dementia were estimated using the Kaplan–Meier method.

Linear mixed-effects regression models were performed in STATA 16.0 using repeated measurements of total MMSE. We performed transformation as described by Philipps et al^32^ to minimize bias due to the ceiling/floor effect and curvilinearity of the raw MMSE score. Time in study, genotype, and an interaction between these were included as fixed effects. Analyses were adjusted for study, age at baseline, sex, and education as fixed effects. All models had patients’ IDs as random intercepts and random slope of time. Marginal predictions of the decline in MMSE were based on fully adjusted models.

Development of PDD was evaluated using Cox regression in R 4.0.4 (package *survival*). The date of PDD onset was computed as the midpoint between the study visit at which dementia was diagnosed and the preceding visit, or as the midpoint between the first record of PDD diagnosis in clinical records or death certificates and the preceding study visit. Patients were censored due to death, loss to follow-up, or last recorded visit. Models were adjusted for confounders: age at baseline, sex, and education. In models including only confounders, Akaike Information Criterion was used to decide on the form of continuous confounders (original, natural logarithm [log] or square root [sqrt] transformed), and interaction with time t (t, sqrt(t), log(t), or log(t + 20)) for variables violating the proportional hazard assumption. If significant, time interaction was added also for genetic variables in the fully adjusted models. PDD-free survival was visualised in Kaplan–Meier plots (package *survminer*). The potential confounding effect of death was investigated using FineGray models allowing for competing risk of death before developing PDD. Data transformed by finegray function was analyzed by weighted Cox regression models adjusted for confounders and time interactions as in the Cox models. All models were stratified for study cohort.

### Standard Protocol and Informed Consent

All participants signed written informed consent and regional ethical committees approved each study.

## Results

### Study Population

The study population is summarized in detail in Supplementary Table 1. A total of 1002 patients were included in the pooled analysis: 139 from CamPaIGN, 146 from ICICLE-PD, 133 from NYPUM, 189 from ParkWest, 250 from PICNICS, and 145 from PINE. The proportion of males was 61.0% (n = 611) and the mean age at diagnosis was 69.1 ± 9.8 years. The median disease duration from diagnosis at baseline was 0.1 years (IQR 0.0–0.2). Patients were followed up for a maximum of 10 years, with a median of 7.2 years (IQR 6.7–10.0). During follow-up, 344 (34.3%) patients died and 177 (17.7%) patients dropped out from the study for reasons other than death. Each of the 1002 patients in the study was genotyped for at least one of *APOE*, *GBA*, *MAPT*, or *SNCA* loci, and 928 (92.6%) had genotype information for all loci (Table 1 and Supplementary Table 3).

### Younger Age at PD Diagnosis in Carriers of APOE-ε4 and GBA Mutations

Carriers of *APOE*-ε4 or any *GBA* mutation were significantly younger at the time of PD diagnosis compared with non-carriers in both unadjusted analysis (Table 1) and after adjustment for sex and study cohort (*APOE*-ε4, β, −2.31; 95% CI, −3.58 to −1.04; *P* < 0.001; *GBA* carriers, β, −2.89; 95% CI, −4.82 to −0.96; *P* = 0.003). Similarly, *APOE*-ε4 and *GBA* were associated with younger age at onset of first motor symptoms (*APOE*-ε4, β, −2.13; 95% CI, −3.43 to −0.83; *P* = 0.001; *GBA* carriers, β, −2.46; 95% CI, −4.38 to −0.53; *P* = 0.012) and at inclusion in the study (*APOE*-ε4, β, −2.39; 95% CI, −3.67 to −1.11; *P* < 0.001; *GBA* carriers, β, −2.95; 95% CI, −4.86 to −1.04, *P* = 0.003), compared to non-carriers. We did not observe any significant differences in age at PD diagnosis for the *MAPT* H1/H2 or rs356219 groups.

### Faster Cognitive Decline in Carriers of APOE-ε4 and GBA Mutations

**FIG. 1.**
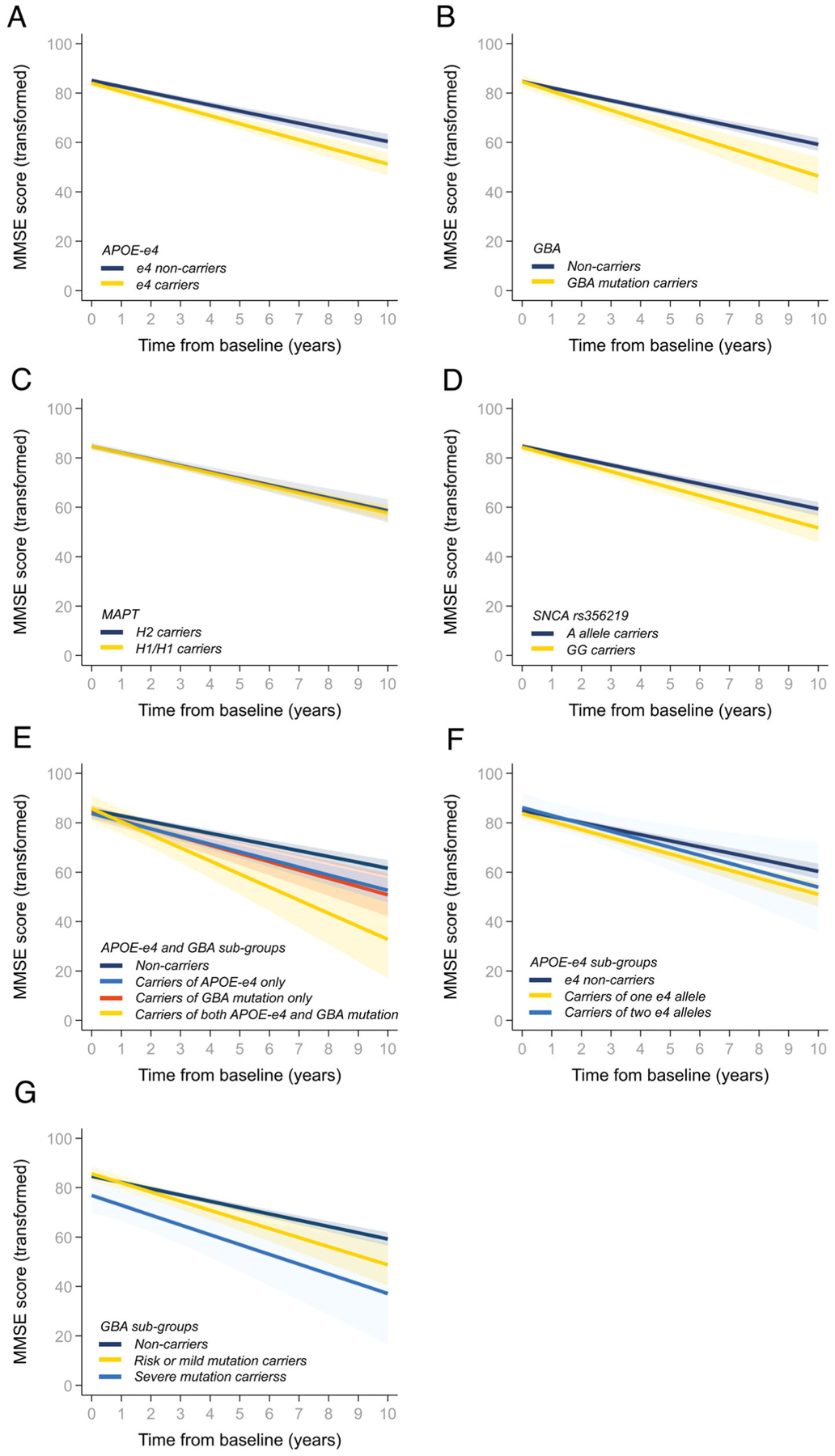
Prediction of Mini–Mental State Examination (MMSE) scores over time. Patients grouped by *APOE*, *GBA*, *MAPT*, and/or *SNCA* genotypes as outlined in the figure keys. MMSE scores were transformed before analysis as described in the Methods.

The rate of annual decline in MMSE score was assessed in linear mixed effects models using data from up to 4477 visits. Both *APOE*-ε4 and *GBA* carriers had a faster rate of annual decrease in scores (Table 2, Fig. 1A and B). For *GBA* carriers, this remained significant after we excluded 6 carriers with variants of unknown severity (interaction with time, β, −1.19; 95% CI, −2.05 to −0.33; *P* = 0.007) or 278 patients screened for selected *GBA* variants using TaqMan and RFLP assays (β, −1.18; 95% CI, −2.16 to −0.19; *P* = 0.019). The estimated drop in MMSE score over 10 years from diagnosis was from around 29 to 24 points for carriers of *APOE*-ε4, and from 29 to 23 points for those carrying any *GBA* mutation, while non-carriers of either *APOE*-ε4 or *GBA* mutations were predicted to decline only to 26 points. An association between rs356219-GG and a faster decline in MMSE score (Table 2; Fig. 1D) marginally missed the threshold for significance after correction for multiple comparisons. No effect on global cognitive decline was shown for the *MAPT* haplotype (Table 2; Fig. 1C).

Based on the significant findings in the primary analysis, we explored the combined effect of harboring both an *APOE*-ε4 allele and *GBA* mutation and found that carriers of both declined faster than carriers of either *APOE*-ε4 or *GBA*, compared to non-carriers (Table 2, Fig. 1E). Over the 10 years, non-carriers were predicted to decline from approximately 29 to 26 MMSE points, while carriers of both *APOE*-ε4 and *GBA* mutations declined to 18 points.

Finally, in secondary analysis, carriers of one *APOE*-ε4 allele were predicted to have a faster annual change in MMSE score, whilst progression in ε4/ε4 carriers was not significantly different compared to non-carriers, though confidence intervals were wide as only 20 participants harbored ε4/ε4 (Table 2, Fig. 1F). Further, both carriers of risk or mild and carriers of severe *GBA* mutations were predicted to experience faster decline in MMSE than non-carriers, although these differences were only significant for those with the risk or mild mutations (Table 2, Fig. 1G).

### APOE-ε4 and GBA Mutations Affect the Progression to PDD

**FIG. 2.**
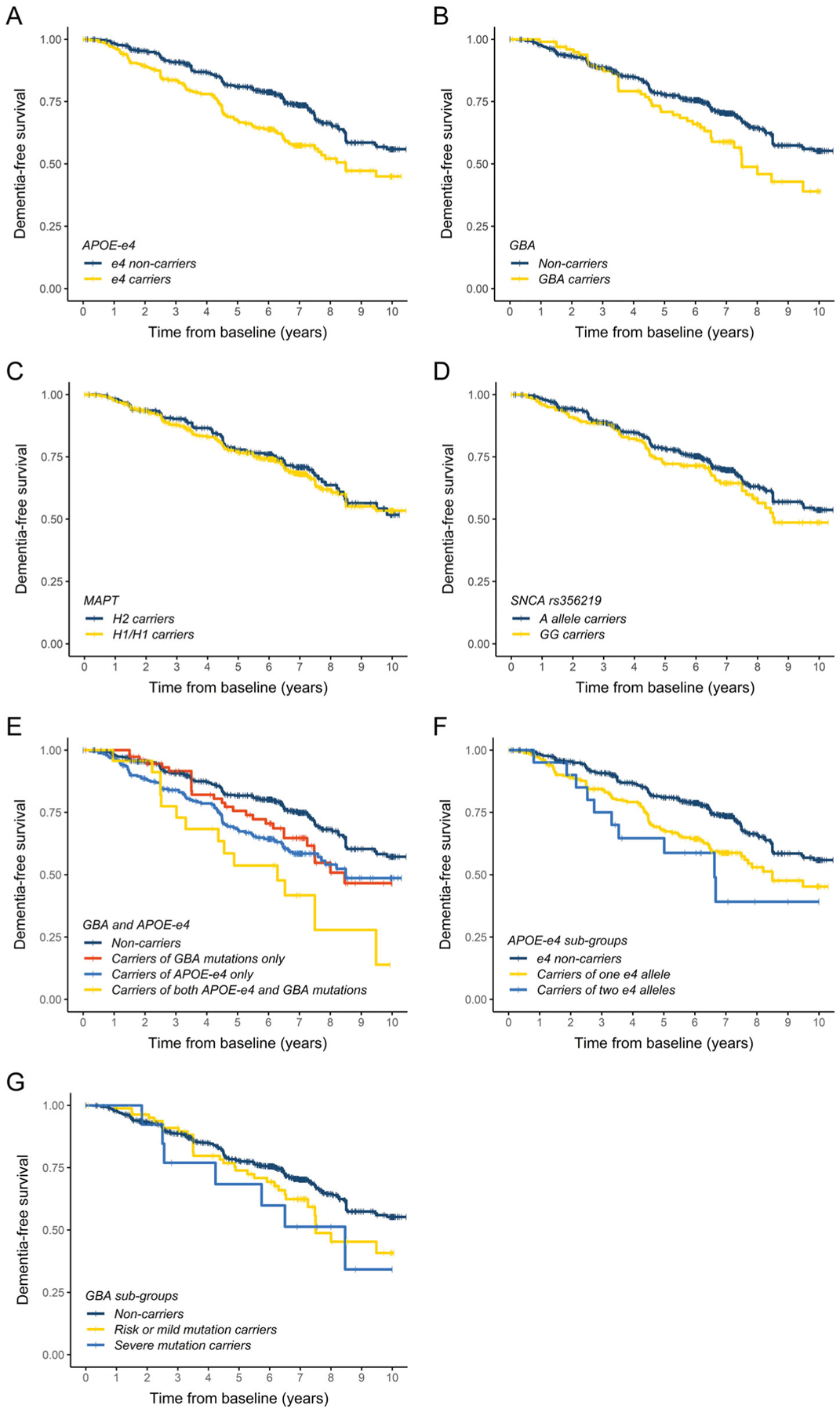
Kaplan–Meier plots of Parkinson’s disease (PD) dementia-free survival. Patients grouped by *APOE*, *GBA*, *MAPT*, and/or *SNCA* genotypes as outlined in the figure keys.

At the study end, 290 of 1002 patients had developed PDD and the cumulative proportion of dementia accounting for deaths and losses to follow-up was 46.7%. *APOE*-ε4 and *GBA* had a significant impact on the rate of progression to PDD, while no effect was observed for either *MAPT* or *SNCA* rs356219 (Table 3, Fig. 2A–D). The model for *APOE* included a significant time-varying effect of the genetic variable (Table 3), which was also found in a competing risk model with death as a competing outcome (Supplementary Table 5) and indicated that the risk of PDD associated with the ε4 allele decreased over time. Patients carrying any *GBA* mutation were at 1.8 times higher risk of dementia (*P* = 0.001), and this remained significant after we excluded the 6 patients with *GBA* variants of unknown severity (HR 1.68; 95% CI, 1.19 to 2.37; *P* = 0.003) or the 278 patients screened for selected *GBA* variants using TaqMan and RFLP assays (HR 1.78; 95% CI, 1.10 to 2.86, *P* = 0.019). Further, carriers of both *APOE*-ε4 and *GBA* mutations were at a 5.2 times higher risk of progressing to PDD than non-carriers (Table 3, Fig. 2E).

Secondary analysis showed a dose-dependent risk of developing PDD associated with *APOE*-ε4: when compared to non-carriers, carriers of one ε4 allele were at 3.1 times higher risk of progressing to dementia, while those who carried two ε4 alleles were at 6.4 times higher risk (Table 3, Fig. 2F). Among the *GBA* subgroups, the risk of developing PDD was 1.6 times higher in carriers of risk or mild mutations, and 2.7 times higher in carriers of severe mutations, when compared to non-carriers (Table 3, Fig. 2G).

## Discussion

Using the largest pooled study of population-based cohorts to date, we have provided data on the impact of genetic variants in *APOE*, *GBA*, *MAPT* and *SNCA* on cognitive decline over the first 10 years of PD. We have shown that both *APOE*-ε4 and *GBA* mutations have an independent and additive effect on cognitive outcomes, adding to mounting evidence that these variants are key drivers of cognitive decline in PD, whilst common variants in *SNCA* and *MAPT* showed no significant impact. Understanding the role of common variants on the pattern of cognitive decline over the natural course of PD will support more accurate disease prognosis and should be considered when designing clinical trials.

Carriers of *APOE*-ε4 were predicted to experience faster cognitive decline, measured using both the annual change in MMSE and the time to a clinical diagnosis of dementia, reinforcing earlier evidence that *APOE* is a major risk factor for cognitive decline in PD^6–8^ as well as other dementias.^4,5^ Further, *APOE*-ε4 had a dose-dependent effect, with about a threefold increased risk of dementia per ε4 allele, comparable to results from a small cohort of neuropathologically confirmed PD cases (HR per ε4 allele 1.82, 95% CI 1.16 to 2.83).^14^ Interestingly, we found that the risk of PDD associated with *APOE*-ε4 was not constant over the course of the disease: carrier status had a large impact on the development of dementia in the early stages of the disease, while no effect of *APOE*-ε4 was seen by 10 years from diagnosis. This may explain why a recent report failed to show an association of *APOE*-ε4 and the development of dementia in a cohort that the authors note was underrepresented for early dementia cases.^33^ Similar observations have also been made in AD, showing the *APOE* genotype–related risk for AD decreases significantly with age,^34^ and would support the importance of early initiation of neuroprotective treatment (when available) in *APOE*-ε4 carriers.

As previously reported,^6^ also by cohorts included in this study,^35,36^ *GBA* carriers were at twofold increased risk of progressing to dementia. This was also reflected in the faster rate of global cognitive decline in patients carrying *GBA* mutations. Further, we show that patients with *GBA* mutations enriched in neuropathic GD progress to dementia faster than patients with *GBA* mutations linked to non-neuropathic GD or risk of PD, compared to non-carriers. Previous studies have used different classification schemes for *GBA* variants, but consistently show that the risk of cognitive impairment increases with the severity of *GBA* mutation.^35,37,38^ Contrary to our findings, several longitudinal studies showed no association with the *GBA* PD-risk mutations, although these were either small studies^39^ or grouped E326K and T369M together with synonymous or intronic variants,^7,40^ potentially diluting their effect. Our study reinforces previous findings^35,41,42^ that not only severe *GBA* mutations, but also the PD-risk variants are important players in modifying the cognitive progression in PD and contribute to the clinical heterogeneity among *GBA* carriers. Given that E326K/T369M are the commonest *GBA* mutations, this knowledge is relevant for a large *GBA*-PD subpopulation. We also show that the severe (neuropathic GD) *GBA* mutations were associated with a greater degree of cognitive impairment at baseline, which indicates that the clinical continuum linked to *GBA* mutations is apparent already at diagnosis.

Lastly, we observed that the risk of cognitive impairment was further increased in individuals harboring both a *GBA* mutation and the *APOE*-ε4 allele. This small subgroup was at fivefold increased risk of progressing to PDD compared to non-carriers. A similar trend was previously observed in a study of 298 patients with PD, where 3 of 6 carriers of both *APOE*-ε4 and *GBA* severe mutations progressed to dementia (HR 2.95; 95% CI 0.80 to 10.90).^7^ A faster decline in MMSE in *GBA* carriers with the *APOE*-ε4 allele was also recently shown in 100 Ashkenazi Jewish patients with DLB.^43^ The increased risk of cognitive impairment observed in carriers of both *APOE*-ε4 and *GBA* mutations is possibly due to the combination of neurodegenerative mechanisms mediated by these genotypes. The compromised activity of mutated β-glucocerebrosidase facilitates the accumulation and aggregation of α-synuclein and *APOE*-ε4 exacerbates the brain accumulation and subsequent deposition of amyloid-β.^1^ Interestingly, two recent studies indicated a novel role for *APOE*-ε4 in enhancing the α-synucleinopathy, and particularly the spread of Lewy body pathology,^44,45^ reinforcing the importance of *APOE*-ε4 as a potential therapeutic target in PDD.

Besides its potential impact on cognitive decline in PD, the direct influence of *APOE*-ε4 on α-synuclein pathology could contribute to the earlier age of PD onset in the ε4 carriers observed in our study, and reported previously.^46,47^ Both of these studies used time of self-reported onset of cardinal PD symptoms, and our study reaffirms this finding of a younger age at clinical PD diagnosis in *APOE*-ε4 carriers in a population-based cohort.

Previous reports on *MAPT* and *SNCA* and cognition in PD are mixed. We show a small effect of rs356219 on global cognitive decline before adjustment for multiple comparisons, replicating our previous finding using a subset of 443 patients included in this current work.^30^ A larger, independent cohort will be required to validate this result, but given the small effect size, the impact of this variant is unlikely to be clinically meaningful. Few studies have reported the impact of *MAPT* or rs356219 on the progression to dementia. In keeping with our findings, a longitudinal study of 298 Spanish patients followed retrospectively found no association between *MAPT* or rs356219 and the time to dementia,^7^ and a study of 514 patients showed no association between *MAPT* haplotype and the years from PD motor onset until PDD.^33^ Contrary to these and our results, an association between *MAPT* H1/H1 genotype and dementia onset was found in two previous survival analyses.^14,17^ Both of these were high-quality studies but were notably smaller (129 patients from the CamPaIGN population-based study, included in this work, and an independent sample of 152 neuropathologically confirmed cases). Many others have shown no association between cognitive test performance, cognitive diagnosis, or rate of cognitive decline and the H1 haplotype^15,16,48–51^ or rs356219,^15,52^ and our study provides additional evidence that the *MAPT* H1 haplotype and *SNCA* rs356219 do not play a key role in cognitive decline in PD.

Longitudinal studies represent a gold standard for tracking disease progression but are traditionally hampered by small sample size, short follow-up time, and losses to follow-up. Considering this, our study has notable strengths, presenting data from the largest, population-based sample of mostly incident PD patients to date, with regular follow-up over up to 10 years. Further, the cohorts had uniform design, used standardized diagnostic criteria for PD and PDD, and attrition (18%) was very low. An additional strength is the applied time-to-event analyses that were also performed allowing for competing risk of death. Potential limitations include incomplete screening for *GBA* mutations, although most participants were genotyped for the common variants and the number of rare mutations or complex *GBA* variants, such as Rec*NCi*I, undetected is expected to be modest. Further, we chose MMSE scores to assess global cognition, and alternatives such as the Montreal Cognitive Assessment (MoCA) are more sensitive to mild and domain-specific changes^53^ and may have revealed subtle effects for some loci. Lastly, it will be important to expand this work to consider promising new candidates, such as the *RIMS2* locus from the first genome-wide survival analysis.^6^

Evidence for the impact of common genetic variants on dementia in the general PD population is important for predicting the prognosis of newly diagnosed patients. In our study, 36% of patients were carriers of either *APOE*-ε4 or *GBA*, which places many individuals at risk of a more severe disease course, and the importance of these results is augmented by the additional effect of *GBA* and *APOE* carrier status on reducing the age at disease onset. Use in clinical practice will necessitate more precise estimates of when patients develop dementia and it will be important to establish the success of combining *GBA* and *APOE* mutations with other predictors. More immediately, the impact of genetic loci on the rate of cognitive decline can be useful in improving clinical trials of putative cognitive neuroprotective agents in PD populations. MMSE is a popular outcome measure in clinical trials due to its short application time and sensitivity to the effect of treatment,^54^ although trials have largely been ineffective, in part attributed to heterogeneity in patient selection and variability in disease progression. Our models estimate that carriers of either *GBA* or *APOE* variants will decline on average 0.5 MMSE points per year, whilst carriers of both variants would average 1.1 points decline per year. Although modest, this is substantially higher than estimates of 0.1–0.2 points per year in other longitudinal studies of unselected cohorts,^55–57^ and suggests that inclusion of genetic variants in trial inclusion criteria could improve homogeneity and trial power.

In conclusion, our findings provide evidence for the role of both *GBA* and *APOE* in the rate of cognitive decline in the general PD population. We show that both *APOE*-ε4 and *GBA* mutations are risk factors for cognitive impairment, and the effect of *APOE*-ε4 on PDD risk is greater in early disease, which should be considered when interpreting the current literature and designing future trials. This knowledge may further improve the accuracy of disease prognosis, especially for those with a younger age at onset who are not traditionally identified as of high risk of rapid cognitive decline.

## Supplementary Material

Supplementary material

## Figures and Tables

**TABLE 1:** Comparison of baseline demographic and clinical characteristics with respect to the *APOE*, *GBA*, *MAPT*, and *SNCA* genotype

Clinical variables	*APOE*-ε4 non-carriers	*APOE-*ε4 carriers	*GBA* non-carriers	*GBA* carriers	*MAPT* H2	*MAPT* H1/H1	*SNCA* rs356219-A	*SNCA* rs356219-GG
N (%)	676 (70.6)	282 (29.4)	876 (89.6)	102 (10.4)	298 (30.3)	684 (69.7)	784 (81.2)	182 (18.8)
Male, N (%)	411 (60.8)	172 (61.0)	533 (60.8%)	65 (63.7%)	180 (60.4)	418 (61.1)	475 (60.6)	114 (62.6)
Age at diagnosis, years, mean (SD)	**69.9 (9.8)**	**67.5 (9.4)**	**69.4 (9.7)**	**66.8 (9.7)**	69.8 (10.2)	68.9 (9.6)	69.3 (9.8)	68.6 (9.7)
Age at baseline, years, mean (SD)	**69.7 (9.8)**	**67.3 (9.4)**	**69.2 (9.8)**	**66.6 (9.7)**	69.5 (10.2)	68.5 (9.6)	69.0 (9.7)	68.5 (9.6)
Age at motor symptom onset, years, mean (SD)	**68.1 (9.9)**	**65.7 (9.5)**	**67.6 (9.9)**	**65.3 (9.4)**	67.8 (10.3)	67.0 (9.7)	67.5 (9.8)	67.0 (9.8)
Positive family history, N (%)	73 (10.9)	37 (13.4)	99 (11.5%)	14 (13.9%)	40 (13.7%)	72 (10.7%)	81 (10.5)	28 (15.6)
Education, years, mean (SD)	11.7 (3.5)	12.1 (3.5)	11.6 (3.4)	11.8 (3.5)	11.7 (3.4)	11.9 (3.5)	11.8 (3.5)	11.8 (3.3)
MMSE score, median (IQR)	29.0 (3.0)	29.0 (2.0)	29.0 (3.0)	29.0 (3.0)	29.0 (3.0)	29.0 (2.0)	29.0 (3.0)	29.0 (2.0)
MDS-UPDRS III, mean (SD)	31.6 (13.3)	29.8 (12.3)	31.2 (13.2)	30.4 (11.6)	32.0 (13.7)	30.6 (12.8)	30.7 (12.8)	32.5 (14.1)
Hoehn and Yahr, median (IQR)	2.0 (1.0)	2.0 (1.0)	2.0 (1.0)	2.0 (1.0)	2.0 (1.0)	2.0 (1.0)	2.0 (1.0)	2.0 (0.6)

Between-group differences marked in bold if *P* < 0.05 in adjusted analysis (multivariate linear regression as described in Methods).

Abbreviations: N, number; SD, standard deviation; IQR, interquartile range; MMSE, Mini-Mental State Examination; MDS-UPDRS III, Movement Disorder Society Unified Parkinson’s Disease Rating Scale Part III; *GBA*, glucocerebrosidase gene; *APOE*, apolipoprotein E gene; *MAPT*, microtubule-associated protein tau gene; *SNCA*, alpha synuclein gene.

**TABLE 2:** Association between the genotype and the annual change in Mini–Mental State Examination (MMSE) score

Genotype	Genotype carriers, N (%)^a^	Main effect^b^ β (95% CI)	*P*	Interaction with time^b^ β (95% CI)	*P*
*APOE-*ε4					
ε4 Non-carriers	661 (70.3)	Ref.		Ref.	
ε4 Carriers	279 (29.7)	−1.07 (−2.90–0.75)	0.249	−0.81 (−1.40–−0.21)	0.008
*APOE*-ε4 subgroups^c^					
Carriers of one ε4 allele	259 (27.6)	−1.25 (−3.12–0.63)	0.192	−0.81 (−1.42–−0.20)	0.009
Carriers of two ε4 alleles	20 (2.1)	1.17 (−4.67–7.00)	0.695	−0.76 (−2.69–1.18)	0.443
*GBA*					
Non-carriers	862 (89.7)	Ref.		Ref.	
*GBA* mutation carriers^d^	99 (10.3)	−0.07 (−2.77–2.62)	0.958	−1.28 (−2.12–−0.44)	0.003
*GBA* subgroups^c^					
Risk or mild mutation carriers	79 (8.3)	0.97 (−2.00–3.94)	0.523	−1.14 (−2.06–−0.22)	0.015
Severe mutation carriers	14 (1.5)	−7.79 (−14.66–0.91)	0.026	−1.43 (−3.63–0.77)	0.202
*MAPT*					
H2 carriers	293 (30.4)	Ref.		Ref.	
H1/H1 carriers	671 (69.6)	−0.03 (−1.81–1.76)	0.978	−0.09 (−0.68–0.50)	0.766
*SNCA* rs356219					
A-allele carriers	771 (81.3)	Ref.		Ref.	
GG carriers	177 (18.7)	−0.46 (−2.58–1.65)	0.668	−0.72 (−1.41–−0.03)	0.041
*APOE*-ε4 and *GBA* subgroups					
Non-carriers of *APOE*-ε4 and *GBA* mutations	574 (62.2)	Ref.		Ref.	
Carriers of both *APOE*-ε4 and *GBA* mutations	23 (2.5)	0.83 (−4.70–6.37)	0.769	−2.87 (−4.46–−1.28)	< 0.001

Models adjusted for study cohort, age at baseline, sex and education.

a Numbers include participants with information for education and Mini-Mental State Examination (MMSE).

b Main effect indicates the effect of genotype on the intercept and the interaction with time indicates the effect of genotype on slope (annual change in MMSE score). MMSE scores were transformed before analysis as outlined in Methods.

c The models of *APOE*-ε4 or *GBA* subgroups include non-carriers of *APOE*-ε4 or *GBA* mutations as the reference group, respectively.

d *GBA* carriers include carriers of any *GBA* mutation, including variants of unknown significance.

Abbreviations: N, number; CI, confidence interval; Ref., reference; *GBA*, glucocerebrosidase gene; *APOE*, apolipoprotein E gene; *MAPT*, microtubule-associated protein tau gene; *SNCA*, alpha synuclein gene.

**TABLE 3:** Cox regression analysis evaluating the effect of genotype on the development of dementia

Group	Total PD, N^a^	PDD, N (%)	Adjusted HR (95% CI)	*P*
*APOE*-ε4				
ε4 Non-carriers	665	169 (25.4)	Ref.	
ε4 Carriers	281	109 (38.8)	3.57 (2.15–5.93)^b^	<0.001
*APOE* ⋆ time			0.88 (0.79–0.99)^b^	0.028
*APOE*-ε4 subgroups^c^				
Carriers of one ε4 allele	261	99 (37.9)	3.14 (1.96–5.02)^b^	<0.001
Carriers of two ε4 alleles	20	10 (50.0)	6.39 (2.53–16.12)^b^	<0.001
*APOE* ⋆ time			0.91 (0.82–0.99)^b^	0.047
*GBA*				
Non-carriers	867	239 (27.6)	Ref.	
*GBA* mutation carriers^d^	100	42 (42.0)	1.76 (1.26–2.46)	0.001
*GBA* subgroups^c^				
Risk or mild mutation carriers	81	32 (39.5)	1.56 (1.07–2.26)	0.020
Severe mutation carriers	13	7 (53.8)	2.71 (1.26–5.82)	0.011
*MAPT*				
H2 carriers	294	81 (27.6)	Ref.	
H1/H1 carriers	676	200 (29.6)	1.17 (0.90–1.52)	0.248
*SNCA rs356219*				
A-allele carriers	775	217 (28.0)	Ref.	
GG carriers	181	62 (34.3)	1.21 (0.91–1.60)	0.199
*APOE*-ε4 and *GBA* subgroups				
Non-carriers of *APOE*-ε4 and *GBA* mutations	577	139 (24.1)	Ref.	
Carriers of both *APOE*-ε4 and *GBA* mutations	23	14 (60.9)	5.19 (2.88–9.38)	<0.001

Models adjusted for sex, age at baseline, education and stratified for study cohort.

a Numbers include participants who had information available for education and time to event or censoring after the baseline visit.

b Model includes interaction between the *APOE* variable and time. The presented HR_*APOE*_ for the *APOE*-ε4 variable refers to the hazard ratio for the respective carriers at time = 0 years (baseline). HR at any point of disease duration can be calculated: HR_*APOE*_ ⋆ (HR_*APOE* ⋆ time_)^t^, where t defines the time point of interest in years.

c The models of *APOE*-ε4 or *GBA* subgroups include non-carriers of *APOE*-ε4 or *GBA* mutations as the reference group, respectively.

d *GBA* carriers include carriers of any *GBA* mutation, including variants of unknown significance.

Abbreviations: PD, Parkinson’s disease; PDD, Parkinson’s disease dementia; N, number; HR, hazard ratio; CI, confidence interval; Ref., reference; *APOE*, apolipoprotein E gene; *GBA*, glucocerebrosidase gene; *MAPT*, microtubule-associated protein tau gene; *SNCA*, alpha synuclein gene.

## Data Availability

Anonymized data that support the findings of this study are available on request and with the correct permissions from the corresponding author. The data are not publicly available due to privacy or ethical restrictions.
