# Supplementary material for: GBA and APOE Impact Cognitive Decline in Parkinson’s Disease: A 10-Year Population-Based Study
Source: Mov Disord. Author manuscript; Available in PMC 2022 Aug 9. (PMC9362732; doi:10.1002/mds.28932)
Supplement: Supplementary material [file NIHMS1817702-supplement-Supplementary_material.docx]

**Supplementary data
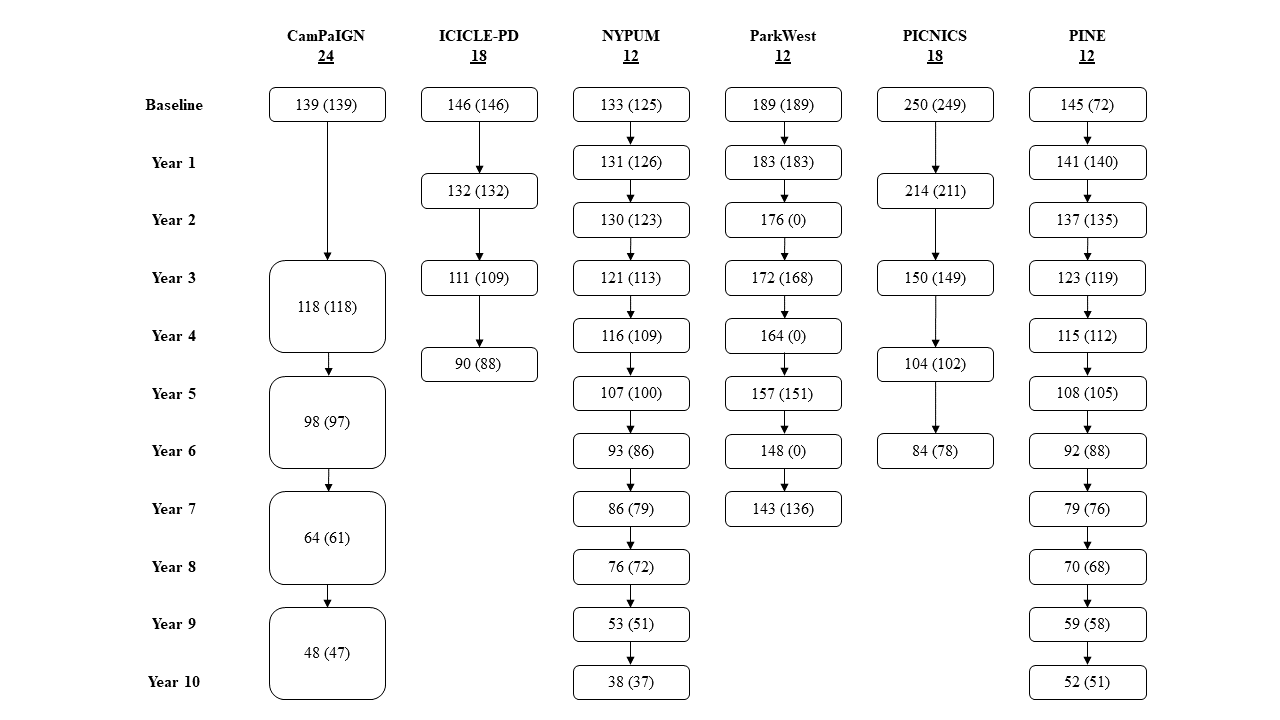
**

**Supplementary Figure 1.** Flowcharts of attendance at the follow-up visits in each cohort. Flowchart refers only to the patients included in this study (i.e. patients with available genetic data). Frequency of visits in months displayed as underscored number. Numbers in the brackets indicate the number of patients assessed by Mini Mental State Examination at the respective follow-up visit.

**Supplementary Table 1.** Description of the six cohorts included in this study and the participants included from each cohort.

|  | **CamPaIGN** | **ICICLE-PD** | **NYPUM** | **ParkWest** | **PICNICS** | **PINE** | **Total** | |
| --- | --- | --- | --- | --- | --- | --- | --- | --- |
| **Cohorts’ characteristics** | | | | | | | |  |
| Country | UK | UK | Sweden | Norway | UK | UK |  | |
| Recruitment period | 1 Dec 2000 – 31 Dec 2002 | 1 Jun 2009 – 30 Dec 2011 | 1 Jan 2004 – 30 Apr 2009 | 1 Nov 2004 – 31 Aug 2006 | Jan 2008 – Oct 2013 | 1 Nov 2002 – 31 Apr 2003; 1 Apr 2006 – 31 Mar 2009 |  | |
| Frequency of follow-up visits (months) | 24 | 18 | 12 | 12^c^ | 18 | 12 |  | |
| Total number of patients in the cohort, N | 140 | 154 | 144 | 191 | 278 | 200 | 1107 | |
| **Characteristics of study population^a^** | | | | | | | |  |
| Patients included in the study, N ^b^ | 139 | 146 | 133 | 189 | 250 | 145 | 1002 | |
| Male, N (%) | 77 (55.4) | 95 (65.1) | 80 (60.2) | 115 (60.8) | 155 (62.0) | 89 (61.4) | 611 (61.0) | |
| Age at diagnosis, years, mean (SD) | 70.3 (9.6) | 65.9 (10.3) | 70.3 (9.6) | 67.9 (9.1) | 68.7 (9.4) | 72.3 (9.9) | 69.1 (9.8) | |
| Age at baseline, years, mean (SD) | 70.1 (9.6) | 66.0 (10.3) | 69.9 (9.7) | 67.6 (9.1) | 68.3 (9.3) | 72.3 (9.9) | 68.9 (9.8) | |
| Age at symptom onset, years, mean (SD) | 68.2 (10.1) | 63.8 (10.3) | 68.6 (9.5) | 65.8 (9.1) | 67.3 (9.5) | 70.6 (9.7) | 67.3 (9.8) | |
| First degree family history, N (%) | 18 (13.3) | 14 (10.1) | 18 (13.5) | 21 (11.2) | 26 (10.6) | 18 (12.4) | 115 (11.7) | |
| Education, years, mean (SD) | 11.4 (3.7) | 12.8 (3.9) | 9.8 (3.8) | 11.1 (3.3) | 12.2 (3.2) | 13.2 (1.8) | 11.8 (3.5) | |
| MMSE score, mean (SD) | 28.0 (2.0) | 29.0 (2.0) | 29.0 (2.0) | 28.0 (2.0) | 29.0 (2.0) | 29.0 (2.0) | 29.0 (3.0) | |
| MDS-UPDRS III^d^, mean (SD) | 34.0 (14.8) | 26.7 (12.0) | 34.1 (13.6) | 30.4 (13.3) | 30.7 (11.9) | 31.2 (12.9) | 31.0 (13.2) | |
| Hoehn and Yahr, mean (SD) | 2.0 (1.0) | 2.0 (0.0) | 2.0 (0.5) | 2.0 (1.0) | 2.0 (1.0) | 2.0 (1.0) | 2.0 (1.0) | |
| Duration of follow-up, years |  |  |  |  |  |  |  | |
| mean (SD) | 5.6 (3.1) | 3.5 (1.6) | 7.2 (2.7) | 6.2 (1.9) | 4.0 (2.4) | 6.8 (3.1) | 5.4 (2.8) | |
| median (IQR) | 5.7 (4.5) | 4.5 (1.6) | 8.0 (5.0) | 7.0 (0.3) | 4.5 (4.3) | 7.1 (5.3) | 5.7 (3.9) | |
| max | 10.2 | 5.2 | 10.3 | 7.9 | 8.0 | 10.5 | 10.5 | |
| Deceased, N (%) | 62 (44.6) | 33 (22.6) | 60 (45.1) | 37 (19.6) | 61 (24.4) | 91 (62.8) | 344 (34.3) | |
| Loss to follow-up, N (%) | 29 (20.9) | 51 (34.9) | 17 (12.8) | 10 (5.3) | 69 (27.6) | 1 (0.7) | 177 (17.7) | |

^a^ Clinical and demographic variables refer only to the patients included in this study. Missing data: age at symptom onset (CamPaIGN, 1; ICICLE-PD, 11; PICNICS, 2); Education years (CamPaIGN, 5; PINE, 5); MMSE score (NYPUM, 8; PICNICS, 1; PINE, 71); MDS-UPDRS III (CamPaIGN, 1; PICNICS, 4; PINE, 4); and Hoehn and Yahr (PINE, 2)

^b^ N, number of patients with PD for which genotyping for *APOE*, *GBA*, *MAPT* or *SNCA* was performed.

^c^ Data collected at the 12 month visits was used in this study; clinical assessments in ParkWest study are performed every 6 months.

^d^ MDS-UPDRS part III scores were converted from UPDRS part III scores as outlined in Methods for CamPaIGN, NYPUM, ParkWest and PINE.

Abbreviations: N, number; SD, standard deviation; MMSE, Mini Mental State Examination; MDS-UPDRS III, Movement Disorder Society Unified Parkinson’s Disease Rating Scale part III; CamPaIGN, Cambridgeshire Incidence of Parkinson’s disease from General Practitioner to Neurologist; ICICLE-PD, The Incidence of Cognitive Impairment in Cohorts with Longitudinal Evaluation-PD; NYPUM, The New Parkinson Patient in Umeå; ParkWest, The Norwegian ParkWest study; PICNICS, Parkinsonism: Incidence, Cognition and Non-motor heterogeneity in Cambridgeshire; PINE, The Parkinsonism Incidence in Northeast Scotland.

**Supplementary Table 2.** Criteria for Parkinson’s disease dementia diagnosis across studies.

| **Study cohort** | **Definition of Parkinson’s disease dementia** |
| --- | --- |
| CamPaIGN | Dementia was diagnosed on the basis of a MMSE of less than or equal to 24 and fulfilment of the Diagnostic and Statistical Manual of Mental Disorders (DSM)-IV criteria for dementia. |
| ICICLE-PD | Subjective cognitive decline and functional independence were determined by a movement disorder specialist or clinician through semi-structured interviews with participants and/or their carers to enable PDD diagnosis using the MDS criteria^1^ in appropriate cases. |
| NYPUM | PDD was assigned according to MDS criteria^1^ by neurologists experienced in neurodegenerative disorders. The PDD diagnosis required the onset of motor symptoms one year prior to the onset of dementia, and cognitive deficiency severe enough to affect activities of daily living that could not be attributed to motor or other dysfunctions. |
| ParkWest | PDD was assigned according to MDS criteria^1^ by 2 study neurologists with experience in movement disorders and dementias. The final decision was based on all available information, including standardized clinical interviews with patients and informants, neuropsychological test results, and assessment of behavioral features (apathy, mood, hallucinations, delusions, daytime somnolence). A diagnosis of PDD was made when patients displayed cognitive decline during prospective follow-ups (clinical interview, MMSE, and neuropsychological tests), deficits ≥ 2 cognitive domains severe enough to interfere with daily living, and functional impairment not attributable to motor, neuropsychiatric, or autonomic symptoms. |
| PICNICS | Dementia was diagnosed using level 1 MDS dementia criteria, which were operationalized in this cohort using the Addenbrooke’s Cognitive Examination-Revised, tests of semantic and phonemic fluency, and the pentagon copying test as well as interview-based assessments with the patient and caregiver. |
| PINE | Dementia was diagnosed with a DSM-IV definition based on clinical interview by an experienced clinician (study neurologist). We required evidence of progressive cognitive decline (detected from prospective follow-up including serial MMSE or MMP), involving at least two cognitive domains which was limiting daily activities, social or occupational function in the absence of delirium. We did not apply strict cut-off scores from cognitive tests or require formal neuropsychological testing. |

**Supplementary Table 3.** Frequency of *APOE*, *MAPT* and *SNCA* genotypes and *GBA* mutations in the study population.

| **Genotype^a^** | **CamPaIGN^b^** | **ICICLE-PD** | **NYPUM** | **ParkWest** | **PICNICS^b^** | **PINE** | **Total** |
| --- | --- | --- | --- | --- | --- | --- | --- |
| Patients included in the study, N | 139 | 146 | 133 | 189 | 250 | 145 | 1002 |
| ***APOE,* N** | 123 | 140 | 133 | 189 | 228 | 145 | 958 |
| ε4 non-carriers | 90 (73.2) | 91 (65.0) | 100 (75.2) | 128 (67.7) | 168 (73.7) | 99 (68.3) | 676 (70.6) |
| ε4 carriers | 33 (26.8) | 49 (35.0) | 33 (24.8) | 61 (32.3) | 60 (26.3) | 46 (31.7) | 282 (29.4) |
| Carriers of one ε4 allele | 31 (25.2) | 46 (32.9) | 31 (23.3) | 57 (30.2) | 51 (22.4) | 46 (31.7) | 262 (27.3) |
| Carriers of two ε4 alleles | 2 (1.6) | 3 (2.1) | 2 (1.5) | 4 (2.1) | 9 (3.9) | 0 (0.0) | 20 (2.1) |
| ***GBA^c^*, N** | 131 | 136 | 133 | 189 | 246 | 143 | 978 |
| Non-carriers | 114 (87.0) | 130 (95.6) | 113 (85.0) | 167 (88.4) | 225 (91.5) | 127 (88.8) | 876 (89.6) |
| *GBA* carriers | 17 (13.0) | 6 (4.4) | 20 (15.0) | 22 (11.6) | 21 (8.5) | 16 (11.2) | 102 (10.4) |
| **Risk^d^** |  |  |  |  |  |  | 72 (7.4) |
| E326K | 6 (4.6) | * | 11 (8.3) | 14 (7.4) | 5 (2.0) | 6 (4.2) | 42 (5.0) |
| T369M | 6 (4.6) | 5 (3.7) | 5 (3.8) | 5 (2.6) | 4 (1.6) | 8 (5.6) | 33 (3.4) |
| **Mild^e^** |  |  |  |  |  |  | 10 (1.0) |
| R48W | 1 (0.8) | * | * | * | 0 (0.0) | * | 1 (0.3) |
| N370S | 1 (0.8) | 1 (0.7) | 0 (0.0) | 1 (0.5) | 6 (2.4) | 0 (0.0) | 9 (0.9) |
| **Severe** |  |  |  |  |  |  | 14 (1.4) |
| R257Q | 1 (0.9) | * | * | * | 0 (0.0) | * | 1 (0.4) |
| L444P | 2 (1.8) | * | 4 (3.0) | 1 (0.5) | 1 (0.7) | 2 (1.4) | 10 (1.4) |
| N462K | 1 (0.9) | * | * | * | 0 (0.0) | * | 1 (0.4) |
| R463C | 0 (0.0) | * | * | * | 2 (0.8) | * | 2 (0.5) |
| **Variants of unknown severity** |  |  |  |  |  |  | 6 (0.6) |
| G10S | 0 (0.0) | * | * | * | 2 (0.8) | * | 2 (0.5) |
| Y135C | * | * | 0 (0.0) | 1 (0.5) | * | 0 (0.0) | 1 (0.2) |
| E388K | 0 (0.0) | 0 (0.0) | * | * | 2 (0.8) | * | 2 (0.4) |
| V460L | * | * | 0 (0.0) | 1 (0.5) | * | 0 (0.0) | 1 (0.2) |
| ***MAPT*, N** | 126 | 143 | 133 | 188 | 247 | 145 | 982 |
| H2 carrier | 44 (34.9) | 44 (30.8) | 35 (26.3) | 45 (23.9) | 85 (34.4) | 45 (31.0) | 298 (30.3) |
| H1/H1 carrier | 82 (65.1) | 99 (69.2) | 98 (73.7) | 143 (76.1) | 162 (65.6) | 100 (69.0) | 684 (69.7) |
| ***SNCA* rs356219, N** | 123 | 144 | 133 | 188 | 235 | 143 | 966 |
| A-allele carrier | 96 (78.0) | 110 (76.4) | 106 (79.7) | 164 (87.2) | 198 (84.3) | 110 (76.9) | 784 (81.2) |
| GG carrier | 27 (22.0) | 34 (23.6) | 27 (20.3) | 24 (12.8) | 37 (15.7) | 33 (23.1) | 182 (18.8) |

* The variant was not included or reported by the genotyping methods; not included in calculation of mutation frequency.

^a^ Frequency reported as N (%).

^b^ 112 patients from CamPaIGN and 142 from PICNICS underwent sequencing; 131 patients from CamPaIGN and 246 from PICNICS were screened using the Illumina Multi-Ethnic Genotyping Array ChIP array which did not include L444P *GBA* mutation.

^c^ All *GBA* amino acid substitutions are numbered excluding the 39-residue signal peptide.

^d^ The total number of carriers of PD risk mutations includes one carrier of the complex genotype E326K + T369M and excludes the two carriers of the complex genotypes E326K + R48W and E326K + N370S.

^e^ Carriers of complex genotypes E326K + R48W and E326K + N370S were classified as carriers of mild *GBA* mutations.

Abbreviations: N, number; CamPaIGN, Cambridgeshire Incidence of Parkinson’s disease from General Practitioner to Neurologist; ICICLE-PD, The Incidence of Cognitive Impairment in Cohorts with Longitudinal Evaluation-PD; NYPUM, The New Parkinson Patient in Umeå; ParkWest, The Norwegian ParkWest study; PICNICS, Parkinsonism: Incidence, Cognition and Non-motor heterogeneity in Cambridgeshire; PINE, The Parkinsonism Incidence in Northeast Scotland; *APOE*, apolipoprotein E gene; *GBA*, glucocerebrosidase gene; *MAPT*, microtubule-associated protein tau gene; *SNCA*, alpha synuclein gene.

**Supplementary Table 4.** Characteristics and classification of *GBA* mutations identified in the study population.

| **Amino acid substitution^a^** | **Allele^b^** | **SNP ID** | **Position^c^** | **Classification according to Beutler et al.^2^** | **Evidence from literature^d^** | **Category^e^** |
| --- | --- | --- | --- | --- | --- | --- |
| G10S/G49S | c.145G>A | rs760930573 | 1:155240048 | - | No data on G10S in GD; only detected in PD (29378790) | VUS |
| R48W/R87W | c.259C>T | rs1141814 | 1:155239934 | Mild | Detected in patients with type I GD (28947706, 7655857), including a homozygous carrier (9295080) | Mild |
| Y135C/Y174C | c.521A>G | rs781152868 | 1:155238584 | - | No data on Y135C in GD; only detected in PD (29792872) | VUS |
| R257Q/R296Q | c.887G>A | rs78973108 | 1:155237453 | Severe | Associated with type II GD (12791040), also in a homozygous carrier (33176831) | Severe |
| E326K/E365K | c.1093G>A | rs2230288 | 1:155236376 | Unknown | Associated with increased risk of PD (23225227, 28830825) | Risk |
| T369M/T408M | c.1223C>T | rs75548401 | 1:155236246 | Unknown | Associated with increased risk of PD (27648471, 33209983) | Risk |
| N370S/N409S | c.1226A>G | rs76763715 | 1:155235843 | Mild | Although N370S was occasionally reported in patients with neurological symptoms (8118463, 21384230), it is nearly exclusively associated with type I GD | Mild |
| E388K/E427K | c.1279G>A | rs149171124 | 1:155235790 | - | Conflicting evidence on causal role in PD risk. Found in both controls and PD patients, with no significant difference in frequencies (25249066), also in large study samples (30302829). | VUS |
| L444P/L483P | c.1448T>C | rs421016 | 1:155235252 | Severe | L444P associated with all GD types, mostly type II and III | Severe |
| V460L/V499L | c.1495G>C | rs369068553 | 1:155235205 | - | No data on V460L in GD; detected in controls and PD (19383421, 29792872, 26296077) | VUS |
| N462K/N501K | c.1503C>G | rs755265316 | 1:155235197 | Severe | Detected only in one GD patient with type II GD and genotype N462K/? (9279145) | Severe |
| R463C/R502C | c.1504C>T | rs80356771 | 1:155235196 | Severe | Associated with type I and III GD in compound heterozygotes (8213821, 10796875) and homozygous carriers (12803123, 9279145, 10604154) | Severe |

^a^ Amino acid substitution in glucocerebrosidase protein excluding / including 39-amino acid signal peptide.

^b^ According to glucocerebrosidase transcript NM_000157.4.

^c^ Position on chromosome according to genome assembly GRCh38.p12.

^d^ Relevant publications as PubMed ID (PMID) numbers.

^e^ Risk, variants of unclear pathogenicity in GD and associated with PD risk; mild, variants associated with non-neuronopathic type I GD; severe, variants associated with neuronopathic type II or III GD; VUS, variants of unknown pathogenicity in both GD and PD.

Abbreviations: SNP, single nucleotide polymorphism; PD, Parkinson’s disease; GD, Gaucher disease; VUS, variants of unknown significance.

**Supplementary Table 5.** Competing risks models evaluating the effect of genotype on the development of dementia with death as competing event.

| **Group** | **Total PD, N^a^** | **PDD, N (%)** | **Adjusted SDHR (95% CI)** | ***P*** |
| --- | --- | --- | --- | --- |
| ***APOE*-ε4** |  |  |  |  |
| ε4 non-carriers | 665 | 169 (25.4) | Ref. |  |
| ε4 carriers | 281 | 109 (38.8) | 3.83 (2.27 – 6.48)^b^ | < 0.001 |
| *APOE* * time |  |  | 0.84 (0.75 – 0.94)^b^ | 0.003 |
| ***APOE*-ε4 sub-groups^c^** |  |  |  |  |
| Carriers of one ε4 allele | 261 | 99 (37.9) | 3.36 (2.07 – 5.45)^b^ | < 0.001 |
| Carriers of two ε4 alleles | 20 | 10 (50.0) | 36.2 (13.8 – 95.0)^b^ | < 0.001 |
| *APOE* * time |  |  | 0.86 (0.78 – 0.94)^b^ | 0.002 |
| ***GBA*** |  |  |  |  |
| Non-carriers | 867 | 239 (27.6) | Ref. |  |
| *GBA* mutation carriers^d^ | 100 | 42 (42.0) | 2.40 (1.68 – 3.42) | < 0.001 |
| ***GBA* sub-groups^c^** |  |  |  |  |
| Risk or mild mutation carriers | 81 | 32 (39.5) | 2.17 (1.46 – 3.24) | < 0.001 |
| Severe mutation carriers | 13 | 7 (53.8) | 2.76 (1.27 – 6.02) | 0.011 |
| ***MAPT*** |  |  |  |  |
| H2 carriers | 294 | 81 (27.6) | Ref. |  |
| H1/H1 carriers | 676 | 200 (29.6) | 1.18 (0.90 – 1.55) | 0.221 |
| ***SNCA rs356219*** |  |  |  |  |
| A-allele carriers | 775 | 217 (28.0) | Ref. |  |
| GG carriers | 181 | 62 (34.3) | 1.34 (0.99 – 1.82) | 0.057 |
| ***APOE*-ε4 and *GBA* sub-groups** |  |  |  |  |
| Non-carriers of *APOE*-ε4 and *GBA* mutations | 577 | 139 (24.1) | Ref. |  |
| Carriers of both *APOE*-ε4 and *GBA* mutations | 23 | 14 (60.9) | 2.26 (1.81 – 2.82) | < 0.001 |

Models adjusted for sex, age at baseline, education and stratified for study cohort.

^a^ Numbers include participants who had information available for education and time to event or censoring after the baseline visit.

^b^ Model includes interaction between the *APOE* variable and time. The presented HR*_APOE_* for the *APOE*-ε4 variable refers to the hazard ratio for the respective carriers at time = 0 years (baseline). HR at any point of disease duration can be calculated: HR*_APOE_* * (HR*_APOE_* _* time_)^t^, where t defines the time point of interest in years.

^c^ The models of *APOE*-ε4 or *GBA* sub-groups include non-carriers of *APOE*-ε4 or *GBA* mutations as the reference group, respectively.

^d^ *GBA* carriers include carriers of any *GBA* mutation, including variants of unknown significance.

Abbreviations: PD, Parkinson’s disease; PDD, Parkinson’s disease dementia; N, number; SDHR, sub distributional hazard ratio; CI, confidence interval; *APOE*, apolipoprotein E gene; *GBA*, glucocerebrosidase gene; *MAPT*, microtubule-associated protein tau gene; *SNCA*, alpha synuclein gene.

**Supplementary methods**

*Genetic data collection*

Genetic data was available or acquired for this study (as indicated below) for *APOE*, *GBA*, *MAPT* and *SNCA* using a combination of whole exome sequencing (WES), genotyping arrays, or targeted genotyping. All TaqMan genotyping assays performed in this study were carried out using TaqPath™ ProAmp™ Master Mix (ThermoFisher) and the ABI PRISM 7300 Real-Time PCR System (Applied Biosystems). The final set of genetic data for each cohort is summarized in Supplementary Table 3.

Briefly, the *APOE*-ε4 allele was defined for 958 patients using genotypes at rs7412 and rs429358: targeted genotyping data was available for 700 patients^3-5^ and acquired for 30 patients using TaqMan assays (C___3084793_20, C____904973_10, Thermo Scientific); for 228 patients genotypes were imputed from the Multi-Ethnic Genotyping Array (MEGA) (Illumina) similar to information reported in detail.^6^

Exonic, non-synonymous *GBA* mutations were identified for 978 patients: for 564 patients, data was available from published WES data (441; mutations detected: G10S, Y135C, R257Q, E326K, T369M, N370S, E388K, L444P, V460L, N462K, R463C)^7, 8^ or MEGA array data (377; mutations detected: G10S, R48W, Y135C, R257Q, E326K, T369M, N370S, E388K, V460L, N462K, R463C),^9^ including 254 patients for whom combination of both WES and array data was used. For these patients, the data was highly convergent (99.2%), except for two samples where mutations T369M and R48W were identified on the array, but not by sequencing. For 136 patients, *GBA* data was extracted from HumanCoreExome-12v1-1 (Illumina) array data (mutations detected: T369M, N370S). For 278 patients, TaqMan genotyping data (E326K, T369M, N370S, Y135C and V460L) was available for 254 patients^10^ or acquired as a part of this study (n = 24), and polymerase chain reaction - restriction fragment length polymorphism (PCR-RFLP) data (L444P) was available for 242 patients^10^ and acquired for 36 patients. For PCR-RFLP assay, a fragment including exons 8-11 of the *GBA* gene was amplified using primers 5’-TGTGTGCAAGGTCCAGGATCAG-3’ and 5’-ACCACCTAGAGGGGAAAGTG-3’^11^ and MyTaq™ Mix polymerase (Meridian Bioscience), and the PCR product was digested using *Nci*I restriction enzyme.

*MAPT* H1 and H2 haplotypes were defined for 982 patients using at least one of rs9468 or rs1800547. Data was available for 767 patients,^3, 5^ extracted from published WES data (n = 187),^8^ or obtained for 28 patients using TaqMan assay (C___7563692_10, Thermo Scientific).

*SNCA* rs356219 genotypes were defined for 966 patients. Data was available for 700 patients^3, 5, 12^ or obtained for 31 patients using TaqMan assay (C___1020193_10, Thermo Scientific). For 235 patients, data was extracted from MEGA array.

For primary analysis patients were grouped by genotype: *APOE*, carriers of the ε4 allele versus non-carriers; *GBA,* carriers of one or more non-synonymous *GBA* mutations versus non-carriers; *MAPT*, carriers of the H1/H1 versus H2 haplotype; *SNCA* rs356219, carriers of the GG genotype versus A-allele.

For secondary analysis, carriers of *APOE*-ε4 were subdivided into carriers of one or two ε4 alleles. *GBA* carriers were subdivided into carriers of PD risk mutations (variants of unclear pathogenicity in GD and associated with PD risk: E326K, T369M, mild mutations (associated with non-neuropathic type I GD: R48W, N370S), severe mutations (associated with neuropathic type II or III Gaucher disease (GD): R257Q, L444P, N462K, R463C), or variants of unknown significance (mutations of unknown pathogenicity in both GD and PD: G10S, Y135C, E388K, V460L). The classification of *GBA* mutations was based on Beutler et al.^2^ and recent literature (summarized in Supplementary Table 4). Due to the small number of mild mutations, *GBA* risk and mild mutations were combined in the secondary analysis.

**References**

1. Emre M, Aarsland D, Brown R, et al. Clinical diagnostic criteria for dementia associated with Parkinson's disease. Mov Disord 2007;22(12):1689-1707; quiz 1837.

2. Beutler E, Gelbart T, Scott CR. Hematologically important mutations: Gaucher disease. Blood Cells, Molecules, and Diseases 2005;35(3):355-364.

3. Yarnall AJ, Breen DP, Duncan GW, et al. Characterizing mild cognitive impairment in incident Parkinson disease: the ICICLE-PD study. Neurology 2014;82(4):308-316.

4. Lange J, Lunde KA, Sletten C, et al. Association of a BACE1 Gene Polymorphism with Parkinson's Disease in a Norwegian Population. Parkinsons Dis 2015;2015:973298.

5. Williams-Gray CH, Mason SL, Evans JR, et al. The CamPaIGN study of Parkinson's disease: 10-year outlook in an incident population-based cohort. J Neurol Neurosurg Psychiatry 2013;84(11):1258-1264.

6. Liu G, Peng J, Liao Z, et al. Genome-wide survival study identifies a novel synaptic locus and polygenic score for cognitive progression in Parkinson's disease. Nat Genet 2021.

7. Winder-Rhodes SE, Evans JR, Ban M, et al. Glucocerebrosidase mutations influence the natural history of Parkinson's disease in a community-based incident cohort. Brain 2013;136(Pt 2):392-399.

8. Gaare JJ, Nido GS, Sztromwasser P, et al. Rare genetic variation in mitochondrial pathways influences the risk for Parkinson's disease. Mov Disord 2018;33(10):1591-1600.

9. Stoker TB, Camacho M, Winder-Rhodes S, et al. Impact of GBA1 variants on long-term clinical progression and mortality in incident Parkinson's disease. Journal of neurology, neurosurgery, and psychiatry 2020.

10. Lunde KA, Chung J, Dalen I, et al. Association of glucocerebrosidase polymorphisms and mutations with dementia in incident Parkinson's disease. Alzheimer's & dementia : the journal of the Alzheimer's Association 2018;14(10):1293–1301.

11. Stone DL, Tayebi N, Orvisky E, Stubblefield B, Madike V, Sidransky E. Glucocerebrosidase gene mutations in patients with type 2 Gaucher disease. Hum Mutat 2000;15(2):181-188.

12. Szwedo AA, Pedersen CC, Ushakova A, et al. Association of SNCA Parkinson's Disease Risk Polymorphisms With Disease Progression in Newly Diagnosed Patients. Front Neurol 2020;11:620585.
